# Using proteomics to advance the search for potential biomarkers for preeclampsia: A systematic review and meta-analysis

**DOI:** 10.1371/journal.pone.0214671

**Published:** 2019-04-05

**Authors:** Thy Pham Hoai Nguyen, Cameron James Patrick, Laura Jean Parry, Mary Familari

**Affiliations:** 1 School of BioSciences, University of Melbourne, Parkville, Australia; 2 Statistical Consulting Centre, University of Melbourne, Parkville, Australia; University of North Carolina at Chapel Hill, UNITED STATES

## Abstract

**Background:**

Preeclampsia (PE) is a leading cause of maternal and perinatal morbidity and mortality worldwide. Although predictive multiparametric screening is being developed, it is not applicable to nulliparous women, and is not applied to low-risk women. As PE is considered a heterogenous disorder, it is unlikely that any single multiparametric screening protocol containing a small group of biomarkers could have the required accuracy to predict all PE subgroups. Given the etiology of PE is complex and not fully understood, it begs the question, whether the search for biomarkers based on the predominant view of impaired placentation involving factors predominately implicated in angiogenesis and inflammation, has been too limiting. Here we highlight the enormous potential of state-of-the-art, high-throughput proteomics, to provide a comprehensive and unbiased approach to biomarker identification.

**Methods and findings:**

Our literature search identified 1336 articles; after review, 45 studies with proteomic data from PE women that were eligible for inclusion. From 710 proteins with altered abundance, we identified 13 common circulating proteins, some of which had not been previously considered as prospective biomarkers of PE. An additional search of the literature for original publications testing any of the 13 common proteins using non-proteomic techniques was also undertaken. Strikingly, 9 of these common proteins had been independently evaluated in PE studies as potential biomarkers.

**Conclusion:**

This study highlights the potential of using high-throughput data sets, which are comprehensive and without bias, to identify a profile of proteins that may improve predictions of PE and understanding of its etiology. We bring to the attention of the medical and research communities that the strengths and advantages of using data from high-throughput studies for biomarker discovery would be increased dramatically, if first and second trimester samples were collected for proteomics, and if standardized guidelines for patient reporting and data collection were implemented.

## Introduction

Preeclampsia (PE), described as a syndrome that affects 5–10% of all pregnancies, remains a leading cause of maternal and perinatal morbidity and mortality [1–3]. Although rare, PE can develop into eclampsia, which is responsible for over 50,000 maternal deaths globally per year [4]. Furthermore, PE is known to affect both the mother and child well beyond pregnancy and can lead to a higher risk of subsequent cardiovascular diseases later in life [5]. Currently, the American College of Obstetricians and Gynecologists (ACOG) [6], the International Society for the Study of Hypertension in Pregnancy [7] and the Society of Obstetric Medicine of Australia and New Zealand (SOMANZ) [8] characterize PE by the presence of new onset hypertension (≥140/90 mm Hg) after 20 weeks of gestation, accompanied by at least one end organ dysfunction (e.g. kidney, liver) or fetal growth restriction (FGR). This updated criterion reflects the growing awareness of the heterogeneity of the etiology of PE.

Clinicians predominantly rely on PE risk factors including age (>35 years), previous PE or family history, multiple gestation, BMI (>25 kg/m^2^), ethnicity, and whether assisted reproductive techniques were involved, leading to classification of low or high risk [9]. However, clinical assessment of these risk factors has limited predictive ability [10,11] because it is not applicable to nulliparous women, and does not encompass all women who develop PE, particularly low risk women who are not monitored as frequently. This is underscored by a recent retrospective study in which 3,230 women, of those classified as low risk pregnancies (28.7% of 10 million women), unexpectedly developed eclampsia [12]. Thus, accurate screening for PE early in pregnancy remains a significant challenge.

Although the underlying etiology of PE is not well defined, and the only effective treatment is removal of the placenta, the predominant view is that PE results from impaired placentation including failure of spiral artery remodeling [13,14]. Therefore, many researchers have focused their attention on a hypothesis-driven approach involving factors implicated in angiogenesis, placental pathology and inflammation [14–18].

A recent study by Townsend et al (2018) reported that there are over 90 predictors and 52 prediction models of PE. This includes 1) maternal characteristics (such as age, parity, blood pressure); 2) biomarkers of which there are over 30, and the most commonly studied are PAPP-A, PlGF, sFlt-1, and PP13; 3) ultrasound tests, including uterine artery Doppler and placental vascularization indices [17]. Thus, although several biomarkers, either alone or in combination, predominately PlGF, sFlt-1, PP-13, PAPP-A, have been investigated for identifying pregnancies at risk of developing PE [17,19–22], the false positive rate is 5–10%, and has low detection rates (24.5% - 33.0%). However when biomarkers are combined with uterine artery Doppler and mean arterial blood pressure, the detection rates can be as high as 100% [22–27] but are applicable to early PE only, and the studies often have very low sample sizes, therefore the predictive values need to be interpreted with care [28].

The multiparametric approach by combining uterine artery Doppler and with different combinations of biomarkers (such as PAPP-A, PlGF, Activin A, Inhibin-A and PP13) and with maternal factors has led to improved detection rates for early preeclampsia [11,19,23,29–31]. However, the multiparametric approach is applied to high risk women only, and uterine artery Doppler is only recommended once new-onset hypertension has been detected at or after 20 weeks of gestation [8]. Although multiparametric screening for PE is promising, due to its current limitations, it is not endorsed in clinical practice by ACOG [1] nor SOMANZ [8].

PE is considered a heterogenous disorder [32] and there is now strong evidence that PE is composed of subgroups, which may have different etiologies [15]. Therefore, it is unlikely that any single multiparametric screening protocol containing a small group of biomarkers could have the required specificity and sensitivity to accurately predict all subgroups. This begs the question, whether the search for biomarkers based on previous hypothesis-driven strategies has been too limiting. While this approach to medical research remains important, the hypothesis-generating approach utilizing high-throughput technologies is an unbiased approach that may lead to unanticipated results [33]. This approach also has the potential to provide us with more information about processes and pathways involved in the manifestation of the multifactorial PE syndrome.

Advances in identifying biomarkers has been given new impetus by recent developments in state-of-the art, high-throughput technologies, in particular, proteomics, which provides an analysis of circulating proteins that may be identified in an unbiased and comprehensive manner [34–39]. Through this approach, proteins involved in cellular functions and pathways affected by disease [40,41], as well as putative disease biomarkers and drug targets have been identified [37]. Utilizing proteomic data can ultimately aid in the identification of novel and effective biomarkers, leading to timely diagnosis and well as improved therapeutic strategies.

There are a multitude of different approaches, and advances in all areas of the analytical pipeline in proteomic studies from experimental design, sample preparation and protein separation, mass analyzer instrument used through to analytical and bioinformatics software, and it is generally agreed that results can vary depending on these many steps, particularly the settings and software used to identify peptides and proteins used [42]. While this background suggests caution should be used when assessing proteomic data, ultimately for clinical purposes when evaluating data quality, the focus is essentially about how the mass analyzer data is converted into useful biological knowledge [42].

With this in mind, the aim of this study was to undertake a meta-analysis of published proteomic studies identifying common proteins in PE. The data in this study highlights the enormous potential of proteomics to advance the search for biomarkers for predicting PE.

## Methods

### Electronic search strategy

All articles were retrieved from PubMed, EMBASE and MEDLINE databases. Search terms used on PubMed included “proteomics” with either “fetal growth restriction” or “preeclampsia” with no restrictions. Search terms used on EMBASE and MEDLINE include “proteomics” with either “pre-eclampsia”, “fetal growth restriction”, “uterine growth restriction”, “fetal growth” or “growth restriction”. Restrictions applied on EMBASE and MEDLINE were human studies, research article and English language. In addition, we examined the reference lists of all known primary and review articles to capture articles missed by the electronic searches.

### Inclusion and exclusion criteria

Article duplicates were removed prior to title and abstract screening for eligibility. Initial exclusion criteria included non-original research (literature reviews that did not contain original data collection), unsuitable data (results that involved interventions or were not collected from PE- or FGR-complicated pregnancies), and non-human studies. Studies were included in the full-text screening if either reviewer identified the study as being potentially eligible, or if the abstract and title did not include sufficient information. Full-text articles and their associated supplementary documents were then obtained and screened. Abstracts that did not mention FGR or PE but included women with pregnancy complications that are associated with FGR or PE, were also eligible for full-text screening (this included pre-term birth, gestational diabetes, gestational hypertension, and small for gestational age).

Articles that did not provide a full list of differentially expressed proteins between PE- or FGR-affected pregnancies compared to healthy pregnancies in different maternal tissues or had insufficient detail about timing of sample collection (trimester of pregnancy) were excluded. The list of differentially expressed proteins provided by authors met cut-off criteria (fold-change and/or p-value) as determined for each study.

**Fig 1:**
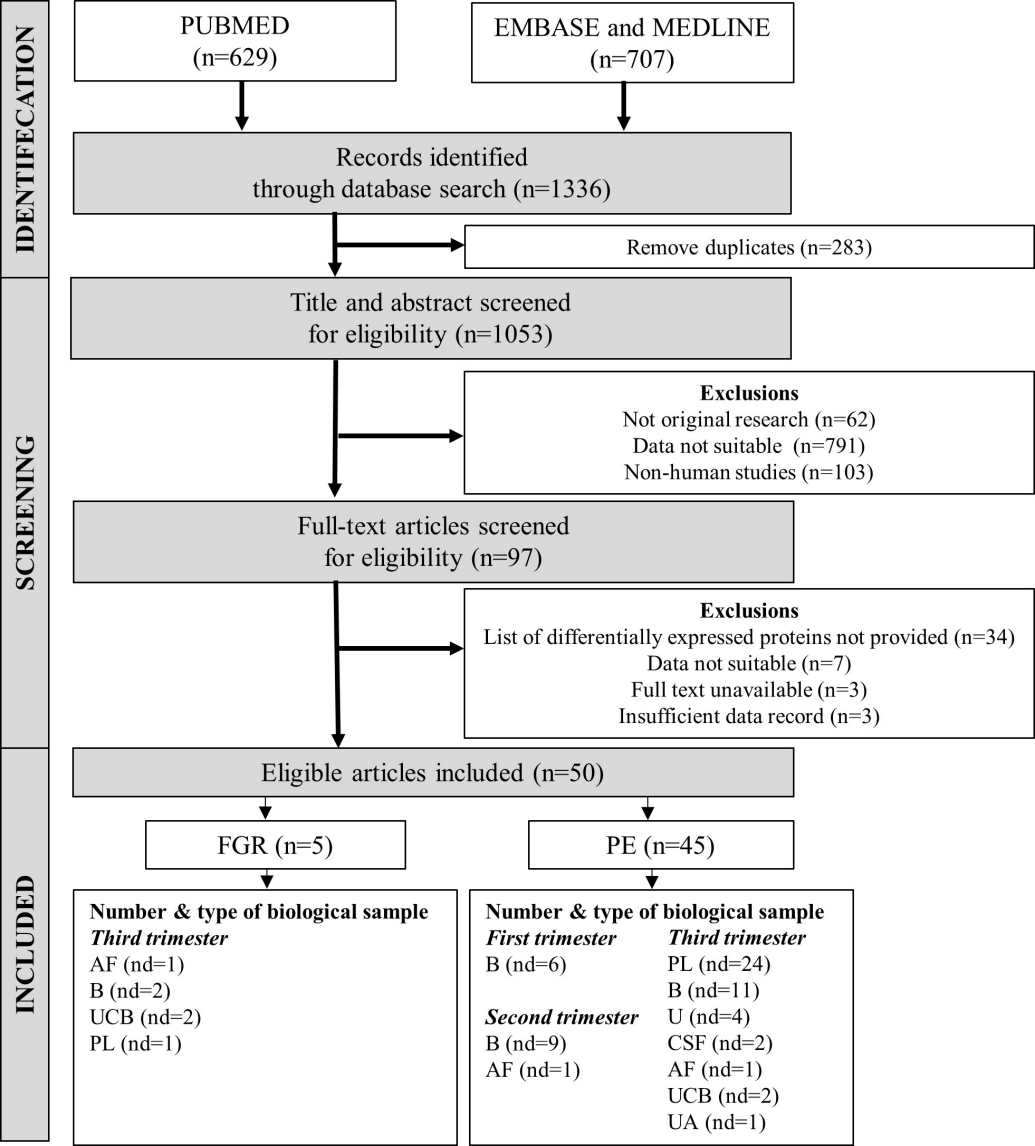
Flow chart showing electronic search strategy used to identify eligible articles for the meta-analysis. Number (n) in brackets refers to the number of articles. Number of datasets (nd) in the eligible articles were grouped according to type of biological sample and the trimester of pregnancy in which the sample was obtained. In some eligible articles, two or more different types of biological samples were used in their studies, giving rise to a total of 6 datasets for FGR and 61 datasets for PE. **Abbreviations:** AF—Amniotic fluid, B—Blood, CSF–Cerebrospinal fluid, PL–Placenta, U–Urine, UA- Umbilical artery, UCB–Umbilical cord blood.

Conference abstracts with no full-text available were also excluded. An outline of the search exclusion and inclusion criteria is shown in Fig 1. The search for articles ended in February 2018 and all articles were evaluated by two independent researchers (T.P.H.N and M.F with < 2% variance) to ensure inclusion and exclusion criteria were strictly met. Disagreements were resolved by consensus. We did not contact authors for further information. The quality of the study population selection and proteomics methodology were evaluated using a modified version of the Newcastle-Ottawa scale [43]; See S1 Table for criteria used for quality assessment.

#### Quality of data assessment

The modified Newcastle-Ottawa Quality Assessment Score **(**See S2 Table) provides an assessment of the quality of the publication and proteomic study reporting but does not address the quality of the proteomic data. Data quality assessment issues in proteomics have been repeatedly addressed [42,44,45], and attempts have been made to adopt and standardize guidelines for reporting proteomic experiments. One of the first initiatives resulted in the ‘Paris Guidelines’ [44] that led to the Journal of Molecular & Cellular Proteomics (MCP) checklist for reporting guidelines (See *www.mcponline.org/site/misc/CheckList.pdf*)

In 2016, proteomic data quality and analysis was the subject of an issue of Proteomics edited by Haynes, Stein and Washburn. In their editorial they suggested, given the multitude of different approaches and advances in all areas of the analytical workflow, ‘…that as long as the data analysis approach used in an experiment is based on sound scientific principles and appropriate fundamental mathematics and statistics, and it is acknowledged that technical changes could affect important considerations, the method should be considered acceptable and the results should be given due consideration.”.

Currently there is not a defined or correct method to approach data analysis in proteomic studies [42]. Therefore, we created a new assessment, which expands on the previous Newcastle-Ottawa Quality Assessment, by taking checklist items, available in the MCP guidelines and re-assessed the 45 eligible papers for the following sections:

1. Experimental Design and Statistics.
2. Protein Identification and False Discovery Rate.
3. Validation.

The criteria address sound experimental design and statistical analysis [42,44], provide a measure of how the protein identification was performed, and whether the search was broad or stringent [46], and validation studies provide context for, and confirmation of the identified differentially expressed proteins [47].

### Population demographics

The selected studies were assessed, and a record was made of the type of tissue used and at what stage of pregnancy samples were collected according to previous definitions [48]: first trimester: <14 weeks; second trimester: 14–28 weeks; third trimester: >28 weeks. Population characteristics recorded included whether patients were smokers, number of participants, BMI, mode of delivery, maternal age, gestational age at delivery, birth weight, ethnicity and country (S1 Table).

Heterogeneity analyses were used to compare–maternal age, gestational age and birth weight–between normal pregnant (NP) and PE or FGR in the selected studies. Some studies included both PE and FGR. In these cases, only the group which was of primary interest in that study was included in the meta-analysis, to avoid double-counting the NP. Data for gestational age reported in days were converted to weeks. The mean, standard deviation and sample size were needed from each study for demographic characteristic comparisons. In some cases, this was not available. Six studies reported medians with range, one study median with standard error of the mean, and one study median with interquartile range (IQR). To pool results in a consistent format, information from these six studies were transformed to sample mean and standard deviation using a mathematical estimator optimised for meta-analyses [49]. Although we maintained high quality standards for the conduct of this systematic review and meta-analysis, which were all matched case-control studies, however they had widely ranging sample sizes. Studies are weighted by the inverse of the variance of the corresponding estimate, so that studies providing more precise estimates are given greater weight. For each outcome, the case effect was calculated as a mean difference (Case–NP), where case is PE or FGR compared to normal pregnancy (NP). A random effects model was used for the meta-analysis, incorporating the possibility of consistent but random variation between studies–for example, due to different locations or study protocols. A statistical test to assess heterogeneity between studies is also included. I^2^ describes the percentage of variability that is due to heterogeneity rather than sample error, and a value of I^2^ > 50% indicates significant heterogeneity among the studies [50]. The analysis was conducted using the ‘meta’ package of R version 3.5.1.

### Identifying differentially expressed proteins in PE and FGR

Direction of change for all differentially expressed protein datasets were obtained from eligible articles. Because proteins have multiple names, isoforms and abbreviations, UniProtKB identification and variations were checked for all differentially expressed proteins to ensure consistency. Proteins that were uncharacterised or did not have UniProtKB identification were excluded from the analyses.

Given the heterogeneity of the human population, and the variability in the preparation and/or detection methods in proteomic techniques, we set the threshold for selecting a common differentially expressed protein for further analysis at ≥ 5 for the PE cohort and ≥ 2 datasets for the FGR cohort. The number of datasets in which a protein was present and direction of change (either upregulated or downregulated) was compiled and used for all further analyses including common differentially expressed proteins between studies for either PE or FGR.

A different approach was used to compare differentially expressed proteins in different biological samples, in order to assess whether changes in one type of tissue were reflected in a different type of tissue. In this approach a cut-off ≥ 2 datasets was also applied.

### Search for original publications testing common differentially expressed proteins

An additional search of the literature for original publications testing any of the common differentially expressed proteins was undertaken. We used the various names of the proteins and “preeclampsia” as search terms. We examined the reference lists of all known primary articles to capture articles missed by the electronic searches. All searches were completed by August 2018. Meta-analysis methods were used to compare expression of common differentially expressed protein between NP and PE in the third trimester. Because of differences in study protocols and concentration levels between studies, mean and standard deviation of circulating proteins were used to calculate standardised mean difference (PE—NP). A random effects model was used as described above.

## Results

### Assessment of eligible proteomic articles

The number of eligible proteomic studies investigating PE and FGR included in this meta-analysis are forty-five and five respectively (Fig 1). As some articles reported data for more than one biological sample, and/or more than one trimester, the total number of datasets available for PE is 61 and for FGR is 6 (See Fig 1)

The PE studies were evaluated by a modified Newcastle-Ottawa Quality Assessment, and a score of ≥ 7 was considered good quality. 82% of studies scored ≥ 7 (total of 10 points; see S2 Table).

As the modified Newcastle-Ottawa Quality Assessment concerns quality of publication, a Quality of data assessment was undertaken. In Supporting Information S3 Table, the scores and the expanded criteria for the 45 eligible studies based on a selection of MCP guidelines are shown. All studies scored 5/5 for experimental design and statistical analysis. Protein identification criteria can be used as a measure of how the search was performed, and how broad or stringent [46]. In this category, there were variable results although all studies listed the differentially expressed proteins that met specific cut-off criteria.

Validation analysis is particularly important for medical research, in order to provide a framework for potential biomarkers. Using bioinformatics for biological analysis has improved immensely and can be a good first step to show whether any differentially expressed proteins identified can be placed in context in pathways and processes [47], before undertaking more time-consuming validations by lab-based approaches such as western blotting or ELISA. Notably, 47% studies provided pathway and processes analysis, while 76% showed lab-based data but in all cases for only a fraction (less than 10%) of the total of differentially expressed proteins identified in any study.

A score of 9 or above was chosen to indicate good quality data. 73% of studies achieved 9 or above, and all included lab-based validation data. Interestingly overall scores were independent of whether a particular journal provided specific reporting guidelines for proteomic experiments. This underscores the variability in reporting proteomic analytical detail, and for this reason we did not exclude any eligible study based on this data quality assessment.

### Population demographics

**Fig 2:**
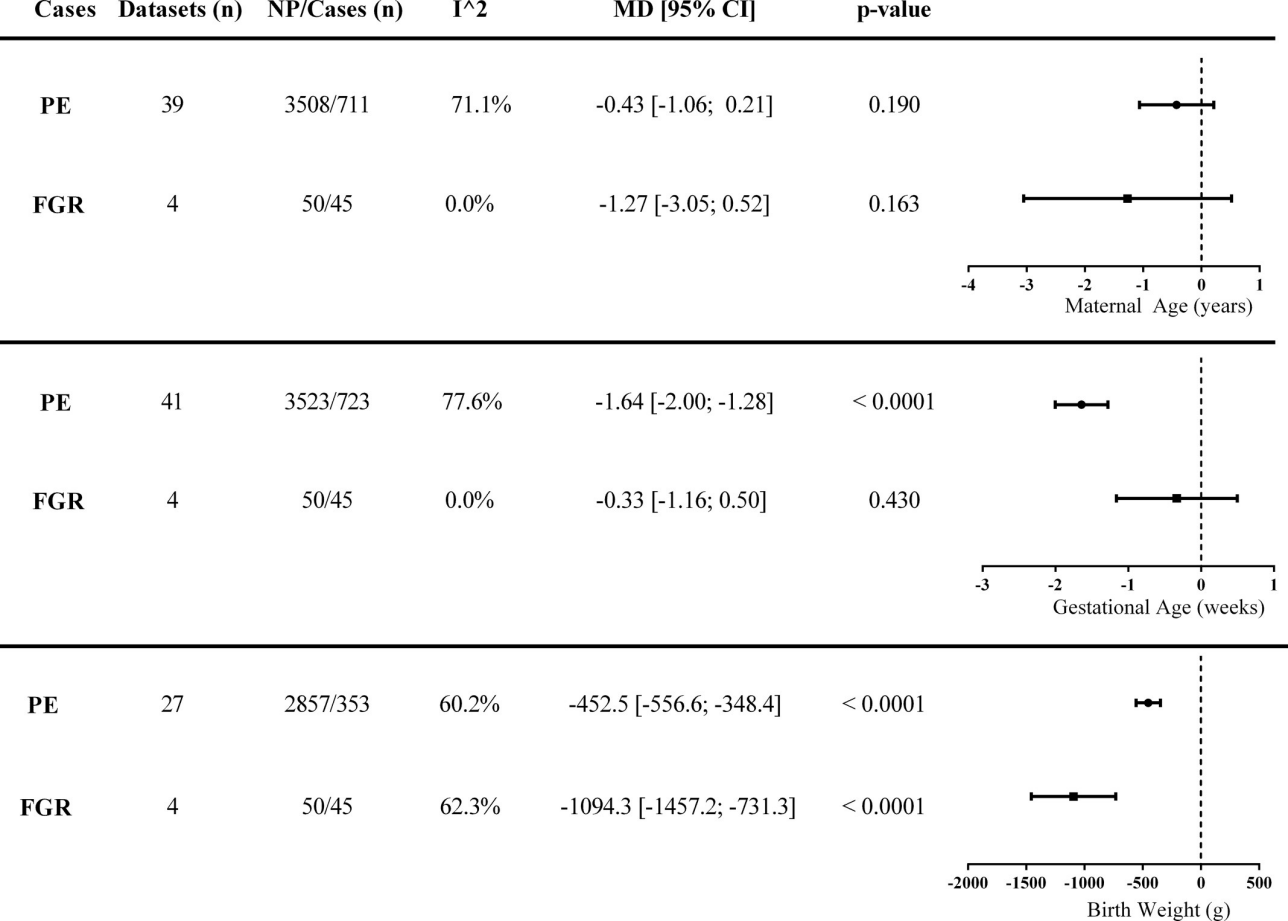
Population heterogeneity in eligible datasets. Maternal age, gestational age and birth weight analyses between preeclampsia (PE), or fetal growth restriction (FGR), compared to normal pregnancies. Heterogeneity (I^2) indicates the percentage of variability within the datasets. Data is presented as mean difference (MD) ± 95% confidence interval (CI). **Abbreviations:** FGR—fetal growth restriction, NP—normal pregnancy, PE-preeclampsia.

Patient characteristics from eligible articles (and their data-sets) included in this meta-analysis are summarized in S1 Table. The number of studies for which characteristics of women (NP versus PE) were compiled is 41 as not all studies provided complete patient demographic data. The results of the meta-analysis of maternal age, gestational age and birth weight are shown in Fig 2.

Maternal age was matched between PE and NP in most studies and was confirmed by the meta-analysis, which showed no significant differences in maternal age between studies (p = 0.190). For PE, the patients were matched for maternal age with NP but gestational age and birth weight are lower (p < 0.0001 for both parameters) as expected [51].

As maternal age was controlled for within studies in the datasets comparing PE and NP, it allowed us to compare the proteomic data. The maximum number of patients for which complete demographic data was available for PE and NP was 723 and 3523 respectively. There was evidence of substantial heterogeneity between PE studies in all measures, with I^2^ > 60%. This is not unexpected and supports the multifactorial etiology of PE.

For FGR, using 4 datasets, the patients were matched for maternal (p = 0.163) and gestational age (p = 0.430), and birth weight was lower (p < 0.0001), as expected compared to NP [52]. There was evidence of heterogeneity in birth weight (I^2^ = 62.3%), whereas maternal (I^2^ = 0%) and gestational (I^2^ = 0%) ages were similar between studies (Fig 2).

### Identification of differentially expressed proteins from proteomic analyses

**Fig 3:**
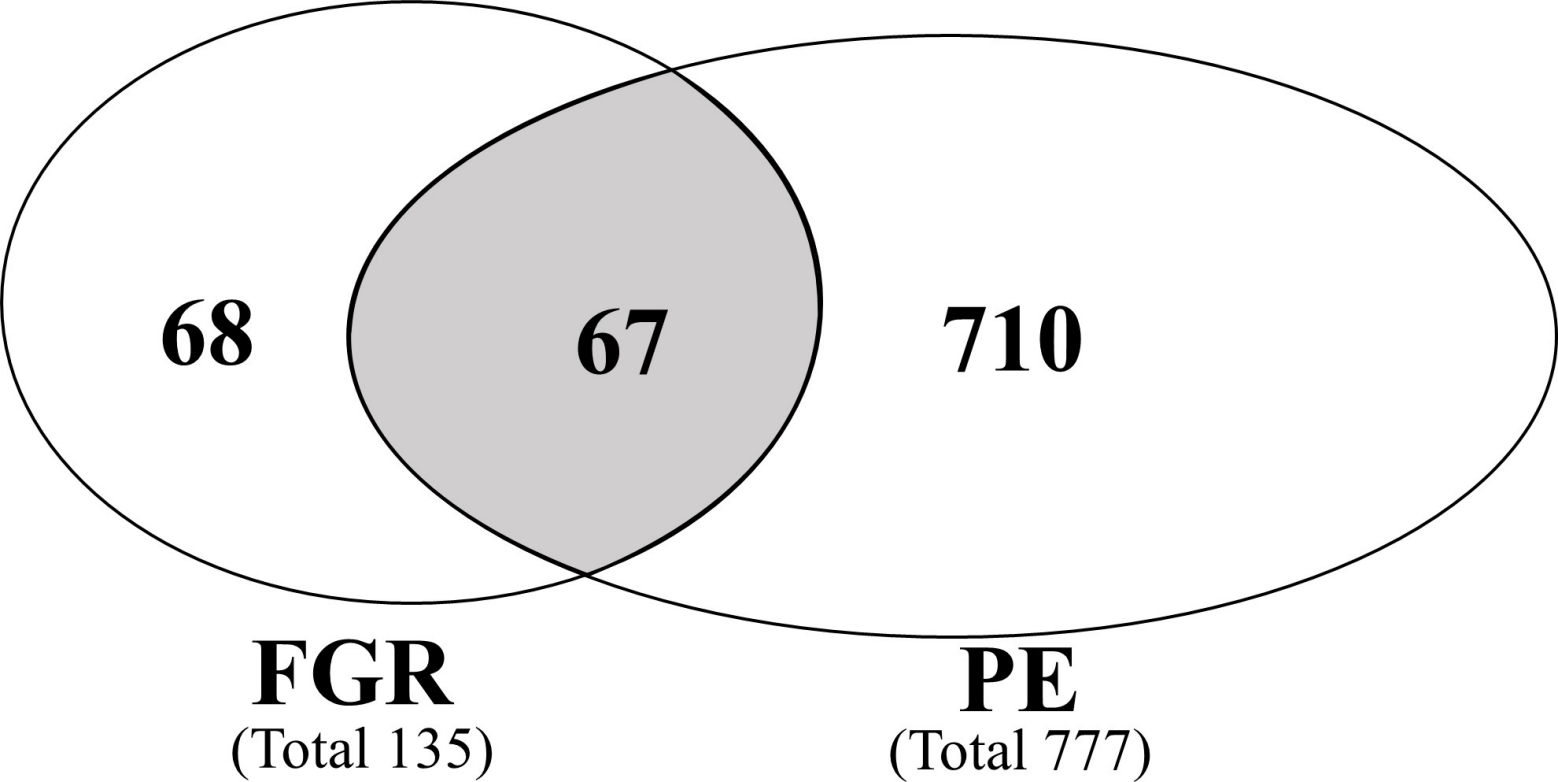
Venn diagram illustrating differentially expressed proteins unique to either fetal growth restriction or preeclampsia, or in common with both pregnancy abnormalities. Diagram shows a total of 845 differentially expressed proteins were identified Fetal growth restriction (FGR) or Preeclampsia (PE) compared to normal pregnancies across different biological samples.

Proteins that were differentially expressed in PE or FGR from each dataset compared to NP were collated. A total of 845 proteins with altered expression were identified across all datasets. 777 and 135 proteins were identified with altered expression in PE and FGR respectively (Fig 3). 67 altered proteins were found to be common to both PE and FGR. S4 Table provides a compilation of the details of all the proteins, including tissue type, trimester of pregnancy and direction of change for these proteins from each dataset. It should be noted that very few proteomic studies (total five datasets) have been published on FGR, according to the search criteria used in this study; and all were in third trimester.

### Identification of common differentially expressed proteins common to PE (cut-off ≥ 5)

To identify proteins unique to PE and FGR, direction of change of all differentially expressed proteins and the number of datasets where it was present were collated in S4 Table. Proteomic technique and validations conducted by all eligible articles are summarized in S5 Table. Given the low number of datasets for FGR (n = 5), further analyses for this condition was not undertaken.

Inclusion criteria for common differentially expressed proteins in PE were presence of a protein in 5 or more datasets (total 45), in any tissue types during the third trimester only. It should be noted that the available datasets for first and second trimester was very low, only 5 and 10 respectively; therefore, we restricted the analysis to differentially expressed proteins during third trimester. 19 common proteins were identified in PE, as listed in Table 1, which includes the number of datasets (n = 5–8), in which the protein expression was altered.

**Table 1:** Differentially expressed proteins common in Preeclampsia during third trimester across all biological samples.

Differentially expressed protein		Present in datasets (n)
**1)**	Alpha 1 Antitrypsin	8
**2)**	Alpha-2-HS-Glycoprotein	5
**3)**	Alpha-Enolase Isoform Alpha	5
**4)**	Ceruloplasmin	5
**5)**	Clusterin	5
**6)**	Fibrinogen Alpha Chain	6
**7)**	Fibrinogen Beta Chain	7
**8)**	Fibrinogen Gamma Chain	5
**9)**	Fibronectin 1	7
**10)**	Gelsolin	5
**11)**	Heat Shock Protein 27	7
**12)**	Hemoglobin Subunit Beta	5
**13)**	Hemopexin	5
**14)**	Histidine-Rich Glycoprotein	5
**15)**	Pregnancy-Zone Protein	6
**16)**	Retinol-Binding Protein 4	6
**17)**	Serum Albumin	7
**18)**	Transthyretin	6
**19)**	Vitamin D-Binding Protein	5

The number of common datasets (n) in which the same protein was listed are shown.

### Identification of common differentially expressed proteins common to PE (cut-off ≥ 2)

Using the cut-off ≥ 2 to analyze the meta-analysis data has led to identification of 130 additional differentially expressed proteins across biological samples, not identified using the previous cut-off ≥ 5. The results of this analysis are tabulated in Supporting information S6 Table.

For almost all of these additional proteins, they were common to, at most, 2 datasets within a biological sample. 66 had consistent direction of change compared to normal pregnancy, while 64 had variable expression. None of these additional proteins were found in all the different types of biological samples. Most proteins were identified in third trimester, predominately in blood and placenta, and had variable expression.

### Analysis of protein changes in different tissues in the third trimester in PE

**Fig 4:**
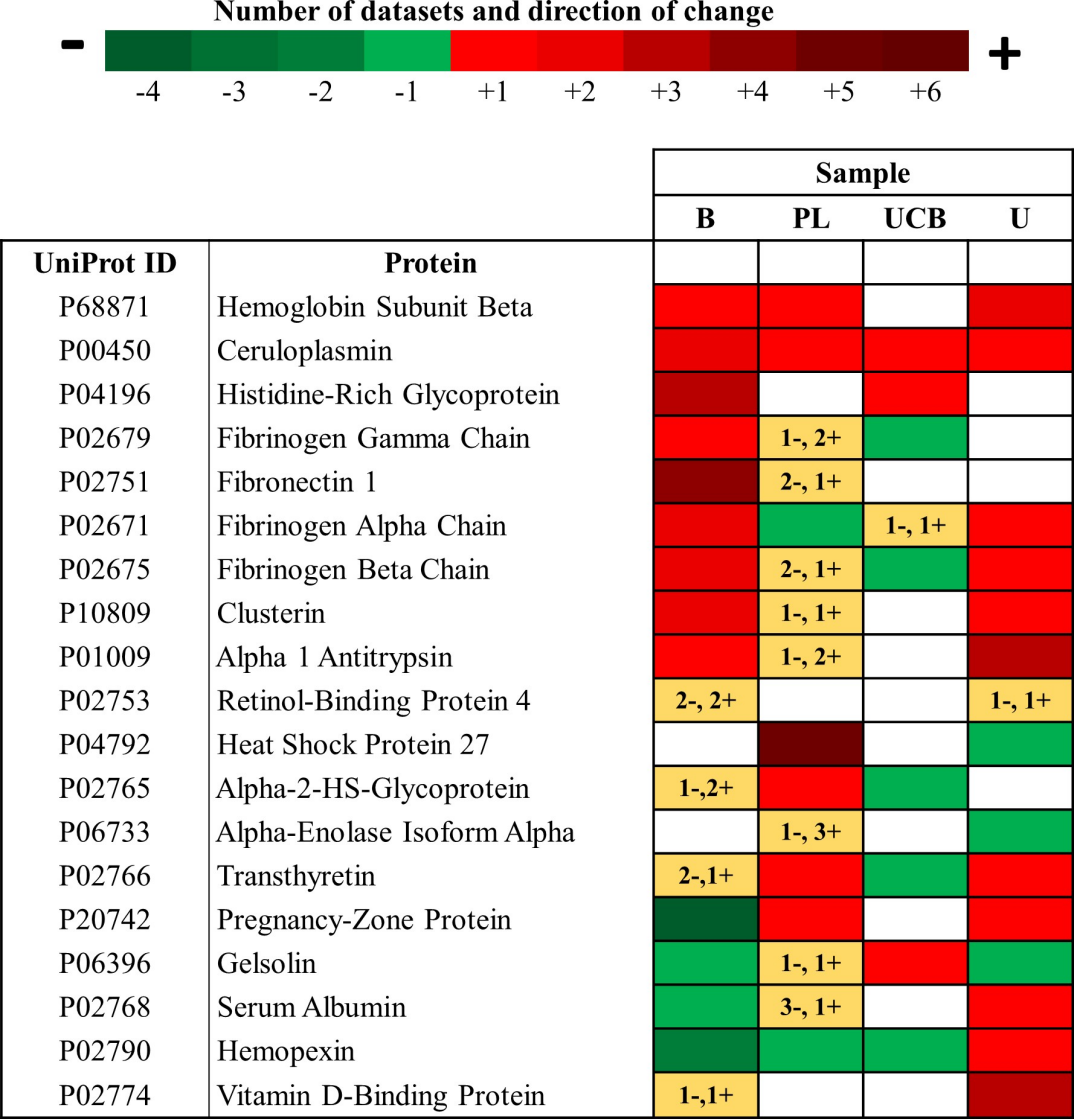
Heat map illustrating differentially expressed proteins in third trimester in Preeclampsia. Altered proteins that were up-regulated (red), down-regulated (green) or had variable direction of change (yellow) across different biological samples are shown. The number of datasets in which each differentially expressed proteins were reported can be determined from the legend. **Abbreviations:** B-blood (including maternal serum and plasma), PL-placenta, UCB-umbilical cord blood, U-urine.

Common differentially expressed proteins during the third trimester in all tissue types in PE compared to NP are presented in Fig 4. For particular proteins in specific tissue types, the direction of change could not be resolved, as both up- and down-expression had been reported for some proteins within datasets. Therefore, these incongruities are represented as yellow squares with the number of datasets listed, and plus and minus symbols to indicate that both directions had been recorded. When comparing common differentially expressed proteins across different tissue types in PE, Hemoglobin Subunit Beta, Ceruloplasmin and Histidine-Rich Glycoprotein were upregulated in all tissues for which data was available. Hemopexin was downregulated in blood, placenta, umbilical cord blood but upregulated in the urine. Fibrinogen Alpha Chain and Clusterin were upregulated in the blood and downregulated in the placenta.

### Circulating differentially expressed proteins throughout pregnancy associated with PE

The existence of pre-symptomatic biomarkers for PE would be of great interest for diagnosis and treatment, particularly if the biomarker could be easily accessed. Proteins derived from biological fluids, such as peripheral blood, which could be obtained throughout pregnancy and particularly before 20 weeks would be ideal as potential biomarkers given ease of access. As the 19 differentially expressed proteins were based on presence in 5 or more datasets across all biological samples, these proteins were examined in only blood across all trimesters. Proteins with consistent direction of change compared to NP were collated in Table 2.

**Table 2:** Profile of protein changes during pregnancy in blood for PE.

Protein name	First Trimester	Second Trimester	Third Trimester
Alpha 1 Antitrypsin	UP	-	UP [1,2]
Clusterin	UP	-	UP[3]
Fibrinogen Gamma Chain	UP	UP	UP
Fibronectin 1	UP[10]	-	UP[4–9]
Ceruloplasmin	-	-	UP[11–16]
Histidine-Rich Glycoprotein	-	-	UP
Hemoglobin Subunit Beta	-	-	UP
Fibrinogen Alpha Chain	DOWN	UP	UP
Fibrinogen Beta Chain	DOWN	UP	UP
Hemopexin	UP	-	DOWN[17–19]
Serum Albumin	-	-	DOWN[20]
Pregnancy-Zone Protein	-	DOWN	DOWN
Gelsolin	-	DOWN	DOWN[21]

Based on presence in 5 or more datasets across all biological samples, the 19 differentially expressed proteins were examined in blood only, across all trimesters. Proteins with consistent direction of change compared to normal pregnancy, irrespective of the number of datasets in which they were present are shown. Superscript numerals indicate the studies that validated particular proteins using non-proteomics techniques and their references are provided. References in table: [1] Espana et al. 1991,[2] Catarino et al. 2012, [3] Shin et al. 2008, [4] Powers et al. 2008, [5] Paternoster et al. 1996, [6] Aydin et al. 2004, [7] Ostlund et al. 2001, [8] Shaarawy et al. 1998, [9] Dogan et al. 2014, [10] Madazli et al. 2005, [11] Niolic et al. 2016, [12] Engin-Unstun et al. 2005, [13] Griffin et al. 1983, [14] Fattah et al. 1976, [15] Guller et al. 2008, [16] Vitoratos et al. 1999, [17] Bakker et al. 2007, [18] Anderson et al. 2018, [19] Gram et al. 2015, [20] Dai et al. 2016, [21] Nadkarni et al. 2016. **Abbreviations:** UP or DOWN refer to up- or down-regulated expression respectively.

As shown in Table 2, some identified common proteins that had altered expression during the third trimester, were also differentially expressed in the first or second trimester. Alpha 1 Antitrypsin, Clusterin, Fibrinogen Gamma Chain, and Fibronectin 1 were upregulated during first and third trimester. Pregnancy-Zone Protein and Gelsolin were both down-regulated in second and third trimester. There were only three proteins for which data was available for all trimesters. These were: Fibrinogen Alpha Chain, Fibrinogen Beta Chain and Fibrinogen Gamma Chain. Fibrinogen Alpha and Beta Chain proteins were downregulated during the first trimester but upregulated in the second and third trimester. However, Fibrinogen Gamma Chain was upregulated throughout pregnancy. Hemopexin, was upregulated during the first trimester and downregulated during the third trimester. There were three upregulated (Ceruloplasmin, Histidine-Rich Glycoprotein, Hemoglobin Subunit Beta) and one downregulated (including Serum Albumin) proteins that were differentially expressed during third trimester but not reported during the first or second trimester.

### Original publications testing common differentially expressed proteins

From an additional search of the literature for original publications testing any of the common differentially expressed proteins in Table 2, 9 of these proteins were independently evaluated in PE during pregnancy. In these previous studies the circulating protein concentration was measured via different assays, such as immunoassay, that is measurement of the protein using a non-proteomic methodology. It is of interest that these 9 proteins, which were considered as potential PE biomarkers in previous studies were also identified in this meta-analysis confirming and validating most of the differentially expressed proteins in the circulation during third trimester. A summary of these 9 proteins examined in independent studies are shown in S7 Table.

**Fig 5:**
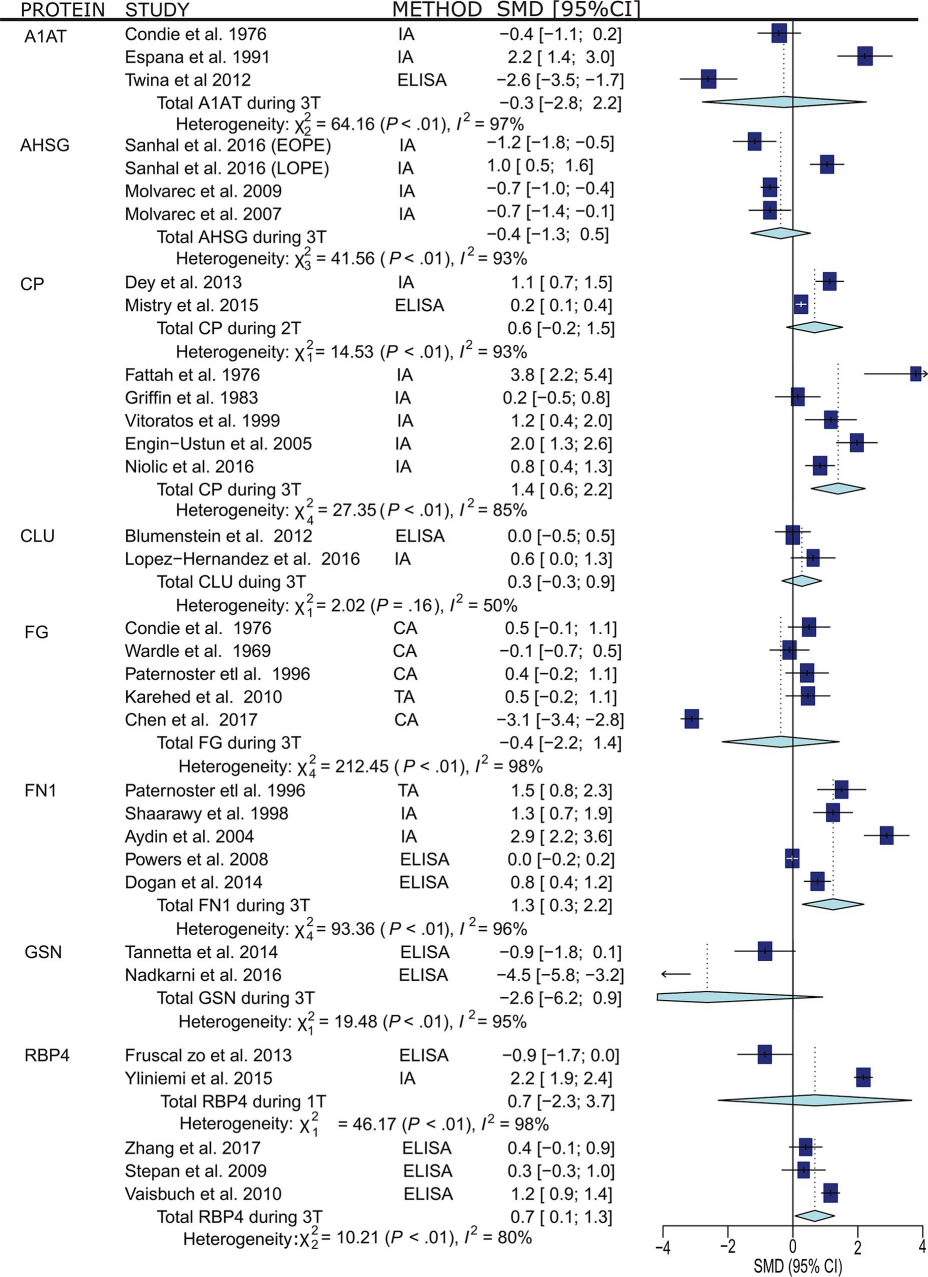
Meta-analyses of candidate differentially expressed proteins examined using non-proteomic techniques to determine the overall direction of change for each protein. **Abbreviations:** 1T- First Trimester, 2T- Second Trimester, 3T- Third Trimester, A1AT- Alpha 1 antitrypsin, AHSG- Alpha 2-HS-Glycoprotein, CA- Clotting assay, CP- Ceruloplasmin, CLU- Clusterin, ELISA- Enzyme-Linked Immunosorbent Assay, FG- Fibrinogen, FN1- Fibronectin 1, GSN- Gelsolin, IA- Immunoassay, RBP- Retinol-Binding Protein 4, SMD- Standardized Mean Difference, TA- Turbidity Assay.

Statistical analysis was applied to the data from these independent studies, where complete data was available, to determine the overall direction of change for each protein, via standard mean difference and the results are presented in Fig 5. Of these 9 proteins, Ceruloplasmin, Clusterin, Fibronectin 1 and Gelsolin, the direction of change was remarkably consistent between this meta-analysis and the independent studies. That is, Ceruloplasmin Clusterin and Fibronectin 1 were upregulated, while Gelsolin was down-regulated in the circulation of women with PE. The expression of Fibrinogen, Alpha 1 Antitrypsin and Histidine-Rich Glycoprotein was consistently increased in the circulation of women with PE from our analysis, however, the results in the independent studies were variable. For Retinol Binding Protein and Alpha-2-HS Glycoprotein, the expression of these proteins was variable in the proteomic studies, but Retinol Binding Protein and Alpha-2-HS Glycoprotein were consistently up-regulated or down-regulated respectively, in the independent studies. While 9 of the 13 differentially expressed proteins have been measured in PE women, the degree of heterogeneity was high, and the data was interpreted with caution.

## Discussion

The current meta-analysis includes data from proteomics studies to date in which NP and PE biological samples were compared and differentially expressed proteins were identified. To our knowledge, this is the first study to compile differentially expressed proteins based on proteomics analyses using a large dataset (3619 healthy pregnant women and 980 preeclamptic women in 45 datasets). First, this study found 19 common differentially expressed proteins in PE, of which 13 were in the circulation of third trimester pregnancies complicated by PE. Second, in an additional search of the literature for original publications testing any of these 13 common differentially expressed proteins in the serum or plasma of PE women during pregnancy, 9 of these proteins have been previously examined and shown to have some association with PE. Thus, validating the proteomic approach.

This meta-analysis, combining the results of high-throughput proteomic data, has broadened the search for circulating biomarkers without bias,. Pregnancy is considered to involve a mild inflammatory response [53,54]. While the majority of the 19 differentially expressed proteins reported here are involved in acute phase responses, all 19 proteins have altered expression in NP. However, they were identified because their expression levels were abnormal in PE, which also involves other cellular changes including oxidative stress and endothelial dysfunction, and some of the 19 proteins (e.g Heat shock protein 27, Gelsolin, Fibrinogen) are involved in these processes [55–57]. To our knowledge, Alpha-Enolase and Pregnancy-Zone Protein have not been validated in non-proteomic studies.

Unfortunately, at present, most of the proteomic data has largely been produced using third trimester maternal samples, and there is a deficit of proteomic studies examining first and second trimester maternal fluids. The list of established biomarkers is currently very limited, and none have been shown to be effective predictors of PE. However, we have shown the potential of proteomics to expand the possible list of possible biomarkers.

### Abnormal levels of secreted proteins in preeclamptic women

There has been extensive research focusing on angiogenic and anti-angiogenic factors (for an example PlGF, sFlt-1 and sEng) as biomarkers to predict the development of PE. While some of these factors have promising predicting values for EOPE only, they have not been implemented into general clinical practice due to low sensitivity and specificity. There are currently several research groups attempting to broaden the biomarker search based on measurement of a subset of markers in the maternal circulation. Allen et al., (2014) systematically examined 10 shortlisted proteins (including sFlt-1, sEng, VEGF and PlGF) in the maternal circulation of women in the first trimester who subsequently developed early or late onset PE. Interestingly only PlGF, PAPP-A, PP-13, sEng and Inhibin A were consistently up-regulated in early onset PE but sFlt-1 and VEGF were not. However, the number of studies from which the data was obtained varied from 1 to 5, reflecting the low number of studies in first trimester in the literature generally, and secondly, while highlighting the established biomarkers, the low number of studies limits the usefulness of these markers in the clinic. While PAPP-A, sEng and VEGF were identified in our meta-analysis using cut-off ≥ 5, these proteins did not meet our threshold for further analyses, as they were common to <5 datasets.

In another systematic review, of 401 identified potential biomarkers before 22 weeks gestation, Wu and colleagues found the efficacy of these markers were inconsistent due to high heterogeneity between studies [58]. These authors concluded that while a single biomarker may be predictive, two or more biomarkers combined with clinical and uterine artery Doppler assessment may be better. Wu et al (2015) also categorized the characteristics of PE (EOPE or LOPE) in line with the definitions from ISSHP [7]. In our study, EOPE and LOPE were not segregated because most studies did not separate these conditions when performing proteomics. A complete list of potential biomarkers identified by Wu et al., (2015) was not available, therefore a direct comparison of their list of biomarkers with the list of differentially expressed proteins identified through proteomic techniques in this meta-analysis (See S4 Table) was not possible.

### Physiological function of candidate differentially expressed proteins during pregnancy

The function of the 9 common differentially expressed proteins identified and validated in this study have well-known functions in the placenta. Alpha 1 Antitrypsin has a cytoprotective role in vascular endothelial cells by suppressing oxidative stress [59], Alpha-2-HS Glycoprotein promotes endocytosis and is involved in trophoblast invasion [60], while Ceruloplasmin is involved in iron and copper transport [61]. Clusterin prevents stress-induced aggregation of blood plasma proteins [62]. Fibrinogen is important in blood clotting [63]. This is of particular interest because Fibrinogen is converted to fibrin clots in response to vascular damage in the placenta [15], the up-regulation of this family of proteins in second and third trimester may be a useful indicator of early placental damage, and perhaps of early onset PE. Fibronectin 1 is involved in cell adhesion and cell motility [64]. Gelsolin is involved in syncytiotrophoblast extracellular vesicle shedding [56]. Hemopexin is involved in the transportation of heme to the liver for breakdown and iron recovery [65]. Retinol-binding Protein 4 mediates retinol transport in blood plasma and is involved in regulating insulin resistance [66]. The functions of these proteins are implicated in the etiology of PE in the placenta. Thus, it is possible that combinations of these 9 differentially expressed proteins may be useful markers for PE.

Although using the cut-off ≥ 2, increased the pool of differentially expressed proteins, most were only present in 2 datasets per biological sample. However, the analysis did highlight a potential profile of proteins that had consistent up- or down-regulation compared to normal pregnancy that were expressed in first or second trimester blood when it would be beneficial to screen pregnant women who may be at risk of developing preeclampsia.

And although 32% of the additional first or second trimester blood proteins that have consistent direction of change are involved in the acute-phase response, [67,68], it may be that a profile of proteins in the first and second trimester, may provide a better prediction for risk, given the complexity and largely unknown etiology of PE.

### Strength and limitations

The major strength of this study is utilizing a large population and segregating the identified differentially expressed proteins based on biological fluid type and gestation from which they were examined. To date there is no effective screening test for PE, which may be attributed to the complex nature of the etiology of PE etiology and its symptoms [15]. As such, examining single candidate biomarkers or combinations of two or three candidate biomarkers, may not reflect the underlying issue of the disease. To have a better overview of the underlying etiology, this meta-analysis pooled a broad range of differentially expressed proteins across different tissues and trimesters of pregnancy. The protein profile approach used in this study encompassed a broader range of proteins. The results were presented from random-effects models allowing heterogeneity in the true effect estimates between different populations and take between-study variation into account. This study applied a strict search strategy and included relevant studies that utilized standard proteomics techniques. This minimized bias by using an explicit, systematic method.

A limitation of this study is that patient characteristics including parity, smoking status, time of sample, medication status and population ethnicity were not sufficiently recorded by all eligible studies in this meta-analysis. Smokers with PE compared to non-smokers with PE have higher levels of ceruloplasmin [69]. In many cases the gestational age at which the samples were collected were not clearly specified. Details regarding medication during pregnancy is important as preeclamptic women, particularly in severe cases, often receive antepartum corticosteroids for fetal lung maturation during birth, which may affect protein secretion in the circulation of the mother [70]. Patients included in this analysis are of mixed background. Therefore, results may vary in populations with a different ethnic background as some angiogenic [71] and renin-angiotensin-aldosterone factors are found to be differentially expressed across different populations [72].

Since this study relied on published articles identified through our search strategies, it is possible that some articles may have been missed, despite our efforts to search through the reference list of already included articles.

As the output of proteomic studies highly depends on a large variety of methods available for each stage of the analytical workflow, including the type of MS instrument and protein sequence database search algorithms used, this can lead to variability in the list of DE proteins identified in the different eligible publications based on proteomic analysis even if using the same tissue.

Currently there are no basic standards for performing proteomic analyses nor are there accepted methods for evaluation of MS-based data quality [42,45]. Our quality of data assessment suggests that the majority (73%) of eligible studies could be accepted as having ‘good data’. This however, does not mean that the remaining studies (27%) did not produce good quality data because it should be noted all studies were original research articles and peer-reviewed, suggesting that all studies met their particular journal’s standard for data quality and reporting.

A criticism of metrics used for data quality assessment is that the values that may be acceptable for one protein identification search tool may not be useful for another, and this means that different parameters are left to the interpretation of the user [73]. Our quality of data assessment criteria were based on our interpretation of a selection of criteria that were useful for our purposes. While replicates were used in all studies, we do not have access to the original data files to determine level of accuracy and reproducibility. And we have already noted that there is huge variability in reporting proteomic analytical detail independent of whether a journal provides guidelines for reporting proteomic studies.

One way to approach evaluation of data quality is to have access to lists of all peptides and proteins identified in all samples by proteomic analysis within each study. Unfortunately, we do not have access to the total list of peptides and proteins identified and quantified by the 45 eligible studies because only lists of differentially expressed proteins were reported.

It is possible to remove high abundance proteins (such as Albumin and IgG) from biological samples, using various methods which has improved the identification of low abundance proteins [74,75]. While around 35% of eligible studies did incorporate a method to depelete high abundance proteins in their proteomic workflow, it is possible that low abundance proteins may not have been detected in some eligible studies, leading to variability in the number and identity of proteins in the different studies.

There is a wide variation in the clinical manifestations and subcategorization of PE, such as early or late onset, term or preterm delivery, maternal or placental origin, mild or severe, and even the presence or absence of FGR. The lack of consistency in the use of PE definition may have introduced additional variability in patient classification. Of the 45 articles included in this meta-analysis, only 4 studies included first trimester samples and 6 studies included second trimester samples. Thus, given our threshold of common proteins in 5 or more datasets, the low number of studies for first and second trimester, makes it challenging to identify common differentially expressed proteins in these trimesters.

### Implications for clinical practice and research

This study has identified 9 proteins that are not part of the usual repertoire for potential biomarkers but have been considered by other researchers to be possible biomarkers for PE. In addition, proteomic techniques for examining altered proteins in biological samples could widen the search for identifying novel effective biomarkers for PE.

Given a current view that PE is composed of subgroups or that these subgroups may have different etiologies [13], it is unlikely that a single biomarker will be an effective screen for the development of PE. This view has also been expressed by others [26,58,76,77] who have also suggested that a profile of biomarkers may lead to better screening, and to enhanced diagnosis of PE subgroups. However, while this study using proteomic data has broadened the range of potential candidate biomarkers, it is largely restricted to data from the third trimester which may not be useful for early prediction of PE. Our work has identified a significant gap in the existing literature, that is the paucity of data for first and second trimester, without which effective biomarkers will remain undetected. Finally, there are currently no consistent guidelines across journals when accepting proteomic data for publication, for authors to include specific patient characteristics nor for data presentation. Future PE proteomic studies should fully enclose patient details, to enable better comparison and meta-analyses. In this way the power of high-throughput data generation can be put to better use.

Using proteomics, a better understanding of the etiologies of the different subgroups of PE may be discovered, which could assist clinicians in tailoring personalized treatments. For example, although aspirin is widely used to prevent pregnancy-related vascular disorders, the mechanisms by which aspirin exerts its action and the indications for its use during pregnancy is an ongoing debate, with a non-response rate of 29–39% in women with a higher risk of PE, preterm birth or of small for gestational age infants [78,79]. A biomarker profile for the different subgroups or early prediction of PE may identify high-risk women who will respond to aspirin treatment. As demonstrated by a recent meta-analyses, aspirin exposure may have greater benefit if started ≤16 weeks of gestation with a daily dose of ≥100mg [80–82]. In future, it might be possible to use proteomics to identify proteins in individual women that are highly responsive to aspirin, allowing lower doses to be used as effective PE prophylactics. At present this approach is being explored for cancer therapeutics [83].

## Conclusion

Broadening the search for a protein profile for specific trimesters of pregnancy by using proteomics is likely to provide a novel approach for improving screening for PE, for understanding of the pathomechanisms of this disorder and its subgroups and most importantly for better outcomes for maternal and neonatal health. Our work has identified a significant gap in the existing literature, that is the paucity of data for first and second trimester, without which effective early onset biomarkers will remain undetected. This review was also limited, by heterogeneity in populations, variations in listed patient characteristics, as well as data-reporting. Detailed standardized reporting criteria is needed to obtain accurate data for systematic review and meta-analyses. Furthermore, high-throughput data collection and large-scale multicenter research that can identify protein profiles rather than a single biomarker for PE is needed to obtain effective screening tools to improve the management of women who will develop PE.

Even within the limitations of this study, 9 differentially expressed proteins, common to PE, were identified in the circulation during third trimester, which were not only examined in other independent studies validating this proteomic meta-analysis approach but has introduced the notion that broadening the potential candidates for screening is worthwhile by using state-of-the art technology.

## Supporting information

S1 PRISMA Checklist The PRISMA Checklist items pertain to the content of a systematic review and meta-analysis. (DOC)

S1 Table Summary of population demographics from eligible datasets. (XLSX)

S2 Table Quality Assessment using a modified Newcastle-Ottawa Scale. Quality of the eligible articles included in the meta-analysis were assessed based on subject selection process, comparability between patients, as well as proteomic study assessment. The selection process for healthy pregnancies (controls) and preeclampsia (PE) were assessed for definition and sample size. Comparability was assessed based on whether the patients were controlled for singleton pregnancies, gestational age and maternal age. Proteomic assessment was based on sample preparation and processing, protein identification and protein quantitation.
(XLSX)

S3 Table Quality of proteomic data assessment. Assessment was based on experimental design and statistics, as well as Protein identification parameters, in which criteria were taken from Molecular and Cellular Proteomics Guidelines (Bradshaw et al., 2006). Whether the differentially expressed proteins were validated using bioinformatics and/or lab-based methods were also included as criteria.
(XLSX)

S4 Table Proteomic dataset compilation. Differentially expressed proteins reported in PE or FGR compared to normal pregnancy, and their direction of change (up-regulated in red, down-regulated in green or variable expression in yellow) in eligible articles were tabulated according to stage of pregnancy and type of biological sample.
(XLSX)

S5 Table Summary of proteomic methodology and proteins validated within eligible studies. Included in this table are the methods of validation and which proteins were validated within the eligible studies.
(XLSX)

S6 Table An analysis of differentially expressed proteins in the eligible studies using cut-off 2 or more. Differentially expressed proteins common in ≥2 datasets across different types of biological samples throughout pregnancy were tabulated. For proteins that were consistently up- or down-regulated, a search of the literature was undertaken to identify any studies in which these proteins had been examined in non-proteomic studies, and these results listed
(XLSX)

S7 Table Independent validation of common differentially expressed proteins. An additional search of the literature was undertaken to identify any studies using non-proteomic techniques in which any of the 19 differentially expressed proteins were examined. The results of this search are tabulated.
(XLSX)

## Abbreviations

EOPE: Early onset preeclampsia
FGR: fetal growth restriction
LOPE: late onset preeclampsia
NP: normal pregnant
PAPP-A: pregnancy-associated plasma protein-A
PE: preeclampsia
PlGF: placental growth factor
sEng: soluble endoglin
sFlt-1: soluble fms-like tyrosine kinase-1
VEGF: vascular endothelial growth factor
